# Large-scale gene co-expression network as a source of functional annotation for cattle genes

**DOI:** 10.1186/s12864-016-3176-2

**Published:** 2016-11-02

**Authors:** Hamid Beiki, Ardeshir Nejati-Javaremi, Abbas Pakdel, Ali Masoudi-Nejad, Zhi-Liang Hu, James M Reecy

**Affiliations:** 1 Department of Animal Science, University College of Agriculture and Natural Resources, University of Tehran, Karaj, 31587-11167 Iran; 2 Department of Animal Science, College of Agriculture, Isfahan University of Technology, Isfahan, 84156-83111 Iran; 3 Laboratory of Systems Biology and Bioinformatics, Institute of Biochemistry and Biophysics, University of Tehran, Tehran, 31587-11167 Iran; 4 Department of Animal Science, Iowa State University, Ames, IA 50011 USA

## Abstract

**Background:**

Genome sequencing and subsequent gene annotation of genomes has led to the elucidation of many genes, but in vertebrates the actual number of protein coding genes are very consistent across species (~20,000). Seven years after sequencing the cattle genome, there are still genes that have limited annotation and the function of many genes are still not understood, or partly understood at best. Based on the assumption that genes with similar patterns of expression across a vast array of tissues and experimental conditions are likely to encode proteins with related functions or participate within a given pathway, we constructed a genome-wide Cattle Gene Co-expression Network (CGCN) using 72 microarray datasets that contained a total of 1470 Affymetrix Genechip Bovine Genome Arrays that were retrieved from either NCBI GEO or EBI ArrayExpress.

**Results:**

The total of 16,607 probe sets, which represented 11,397 genes, with unique Entrez ID were consolidated into 32 co-expression modules that contained between 29 and 2569 probe sets. All of the identified modules showed strong functional enrichment for gene ontology (GO) terms and Reactome pathways. For example, modules with important biological functions such as response to virus, response to bacteria, energy metabolism, cell signaling and cell cycle have been identified. Moreover, gene co-expression networks using “guilt-by-association” principle have been used to predict the potential function of 132 genes with no functional annotation. Four unknown Hub genes were identified in modules highly enriched for GO terms related to leukocyte activation (*LOC509513*), RNA processing (*LOC100848208*), nucleic acid metabolic process (*LOC100850151*) and organic-acid metabolic process (*MGC137211*). Such highly connected genes should be investigated more closely as they likely to have key regulatory roles.

**Conclusions:**

We have demonstrated that the CGCN and its corresponding regulons provides rich information for experimental biologists to design experiments, interpret experimental results, and develop novel hypothesis on gene function in this poorly annotated genome. The network is publicly accessible at http://www.animalgenome.org/cgi-bin/host/reecylab/d.

**Electronic supplementary material:**

The online version of this article (doi:10.1186/s12864-016-3176-2) contains supplementary material, which is available to authorized users.

## Background

The completion of a draft genome assembly simply marks the “end of the beginning” of genome exploration in that species [1]. After a genome is sequenced, the next critical step is gene annotation. This includes marking the genomic position and structure of genes, naming genes (nomenclature) and functional annotation i.e. identifying their biological function. Since the sequencing of the cattle genome in 2009 [2], there have been efforts to identify functional elements in the genome [3–7]. Functional genomics focuses on understanding the function and regulation of genes and gene products on a genome-wide or global scale [1]. Initial annotation of the cattle genome identified 22,000+ genes, with a core set of 14,345 orthologs shared among seven mammalian species [2]. Despite these efforts, the function of some genes is still not understood, or partly understood at best [6].

The large amount of biological data deposited in public databases provides an opportunity to computationally annotate functional and regulatory connections among genes. A challenge in this post-genomic era is to properly integrate available information so as to reconstruct, as accurately as possible, valuable information from large volumes of data [8]. It is widely accepted that to understand gene function, genes must be studied in the context of networks [9]. Gene co-expression analysis (GCA) has emerged as a powerful systems biology approach for multigene analysis of large-scale data sets with functional annotation (the assigning GO term to an identified gene) [9–12]. This technique has been widely used to functionally annotate gene from different species [12–16]. An output of GCA is the ability to annotate gene function based on ‘guilt-by-association’ (GBA). In short, groups of genes that maintain a consistent expression relationship (i.e. co-expression modules) may share a common biological role [11]. The evolutionary conservation of co-expression patterns lends further evidence to support the biological importance of this phenomenon [17].

In this study, a condition independent gene co-expression network was generated to provide additional functional annotation information for genes in the cattle genome. We have annotated genes with possible putative functions and possible regulatory mechanisms. This effort will accelerate discovery of genes and lead to elucidation of the biological features responsible for economic traits. Network information is publically available at http://www.animalgenome.org/cgi-bin/host/reecylab/d.

## Results

**Fig. 1:**
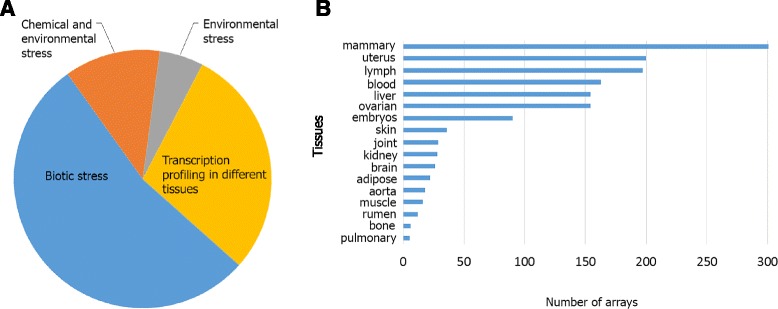
Composition of the 1470 *Affymetrix* Genechip Bovine Genome Arrays used in this study. Arrays were classified according to the experimental conditions (**a**) and distribution (**b**)

**Fig. 2:**
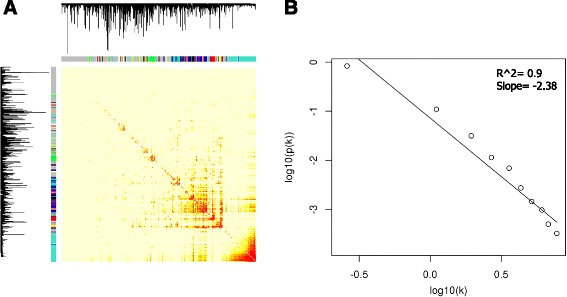
Network topology of the CGCN. **a** Visualizing the CGCN (based on TOM similarity matrix) using heatmap plot. Light color represents low overlap and progressively darker red color represents higher overlap. Blocks of darker colors along the diagonal are the modules. The gene dendrogram and module assignment are also shown along the left side and the top. **b** Scale free topology evaluation of CGCN using Scale-Free Topology Fitting Index [18]

Data from 72 experiments (Additional file 1: Table S1), which equated to 1470 publically available Affymetrix Genechip Bovine Genome Arrays, was used to construct a Cattle condition-free Gene Co-expression Network (CGCN). These experiments covered 17 tissues and four broadly classified experimental conditions (Fig. 1). Only probe sets that mapped to unique Enterz Gene ID’s (16,607 probe sets represented 11,397 genes) were used for gene network construction (Additional file 2: Table S2). Weighted Gene Co-expression Network Analysis (WGCNA) [18] was used to identify highly connected gene sets (modules) based on their normalized expression levels (see Methods). Sixty percent (10,095 of 16,607 probe sets), of probe sets were consolidated into 32 modules (Fig. 2a and Additional file 2: Table S2). The approximate high Scale-Free Topology Fitting Index (*R* ^2^) shows approximate scale free topology in CGCN (Fig. 2b).

**Fig. 3:**
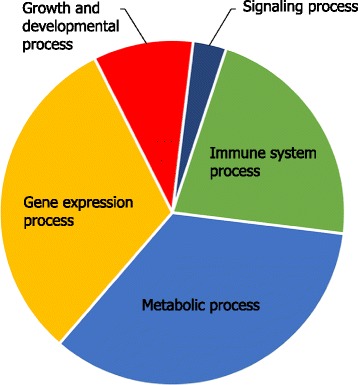
Manual category of ove-represented BP GO terms in CGCN modules

Since genes belonging to the same module are co-expressed across a vast array of tissues and experimental conditions, they are likely to encode proteins with related functions or are within a given pathway [14]. The potential biological function of the identified modules were investigated using gene ontology [19] and Reactome pathway information [20] (functional enrichment analysis). Almost all modules exhibited high enrichment for GO terms (32 modules) or Reactome pathways (29 modules) (Table 1 and Additional file 3: Table S3). The concordance between enriched GO terms and pathways in each module strengthened the biological function of computational modules. The biological function of modules in CGCN can be categorized into four major functional categories (metabolic process, gene expression process, immune system process and growth and developmental process) (Fig. 3). In each module, there were several tight clusters of GO terms that had many links between these groups of genes (Additional file 3: Table S3), which may represent robust interactions between these processes.

**Table 1:** Modules identified in the network and their top over-represented Biological process GO terms

Module	# probes	# genes	GO term	Genes in GO term^a^	*p*–value^b^
Turquoise	2569	2050	system process	241/652	3.30E-31
Blue	806	690	nucleic acid metabolic process	211/2322	6.89E-10
Brown	606	526	organic acid metabolic process	113/565	1.38E-36
Yellow	566	521	RNA processing	83/455	6.26E-26
Green	557	471	RNA metabolic process	151/2064	3.36E-09
Red	519	431	cell cycle process	117/604	4.93E-50
Black	454	384	negative regulation of peptidase activity	22/129	2.76E-07
Pink	348	304	RNA metabolic process	117/2064	7.20E-17
Magenta	292	267	gene expression	111/2568	1.56E-08
Purple	275	361	ribonucleoprotein complex biogenesis	44/266	3.10E-24
Green yellow	271	214	chromatin modification	26/336	3.79E-07
Tan	269	225	anatomical structure morphogenesis	72/1323	9.65E-14
Salmon	239	216	translation	85/398	1.40E-67
Cyan	202	181	oxidation-reduction process	35/619	1.15E-08
Midnight blue	200	191	hydrogen ion transmembrane transport	14/60	1.14E-10
Light cyan	188	151	leukocyte activation	34/365	7.29E-20
Grey60	184	158	leukocyte activation	30/365	3.28E-13
Light green	169	160	purine ribonucleoside triphosphate metabolic process	17/133	2.08E-09
Light yellow	165	149	sterol biosynthetic process	15/31	2.21E-18
Royal blue	164	146	mitochondrial translation	8/43	9.87E-06
Dark red	158	139	proteasomal protein catabolic process	12/245	6.00E-3
Dark green	146	128	response to endoplasmic reticulum stress	15/146	2.23E-09
Dark turquoise	120	88	tricarboxylic acid metabolic process	3/27	5.75E-3
Dark grey	102	80	inflammatory response	28/269	1.40E-23
Orange	98	78	antigen processing and presentation of peptide antigen	12/26	1.00E-18
Dark orange	95	85	regulation of mRNA metabolic process	3/69	1.40E-2
White	80	63	defense response to virus	15/138	4.68E-15
Sky blue	67	53	transcription from RNA polymerase I promoter	3/32	4.91E-4
Saddle brown	62	53	defense response to bacterium	7/94	1.16E-11
Steel blue	61	48	acute inflammatory response	6/46	7.97E-07
Pale turquoise	34	24	L-serine metabolic process	3/7	2.55E-06
Violet	29	23	amino acid activation	7/36	1.84E-12

^a^The two values listed in this column refer to the number of genes associated with the over-represented GO term in the module and the number of genes associated with the same GO term in Affymetrix Genechip Bovine Genome Array

^b^The *p*-value indicated the probability that a module contains equal or larger number of genes associated with the GO term under a hypergeometric distribution after Bonferroni step-down correction

**Fig. 4:**
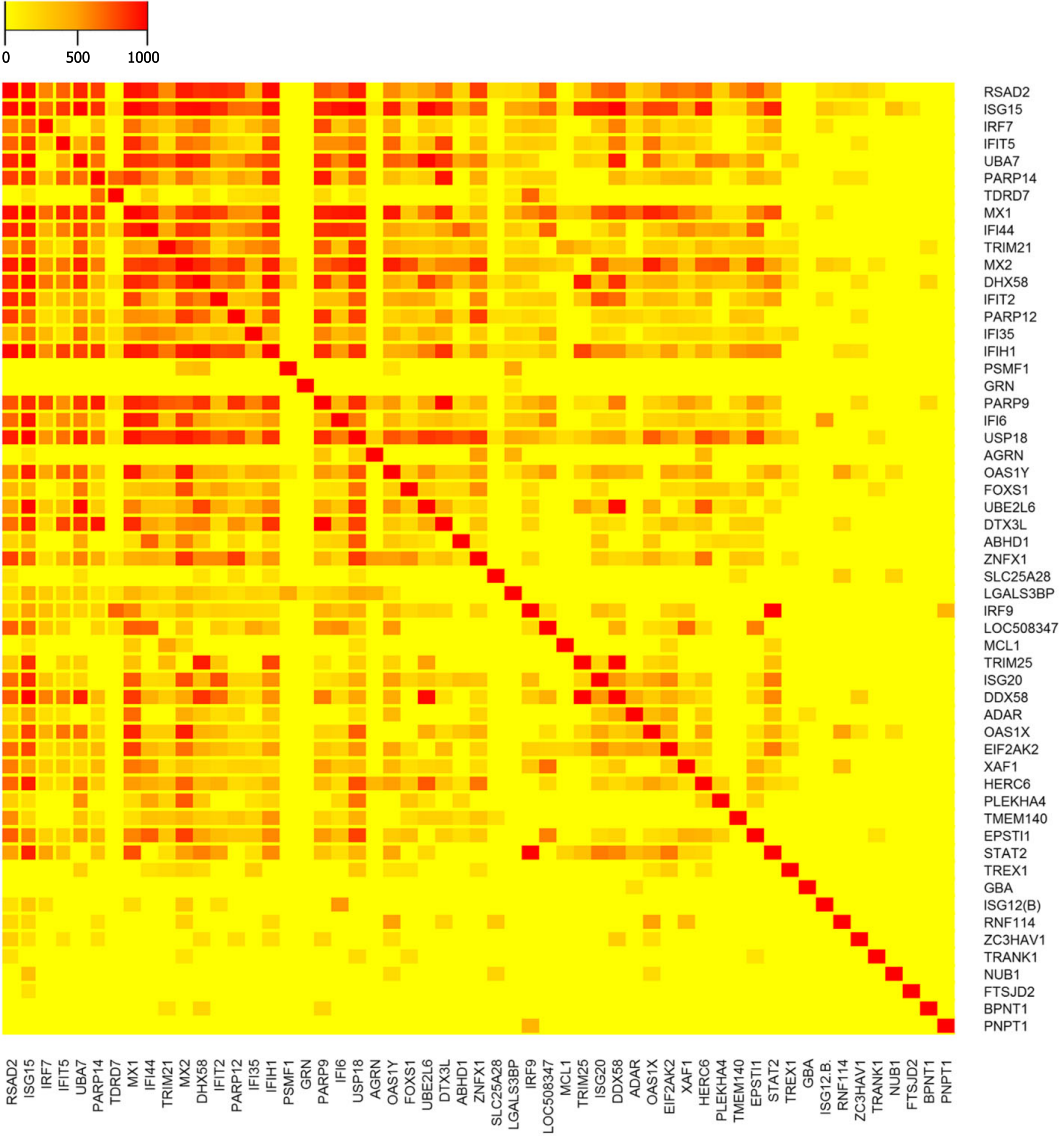
Heatmap visualization of module one gene interactions based on cattle protein interaction network [21]

**Fig. 5:**
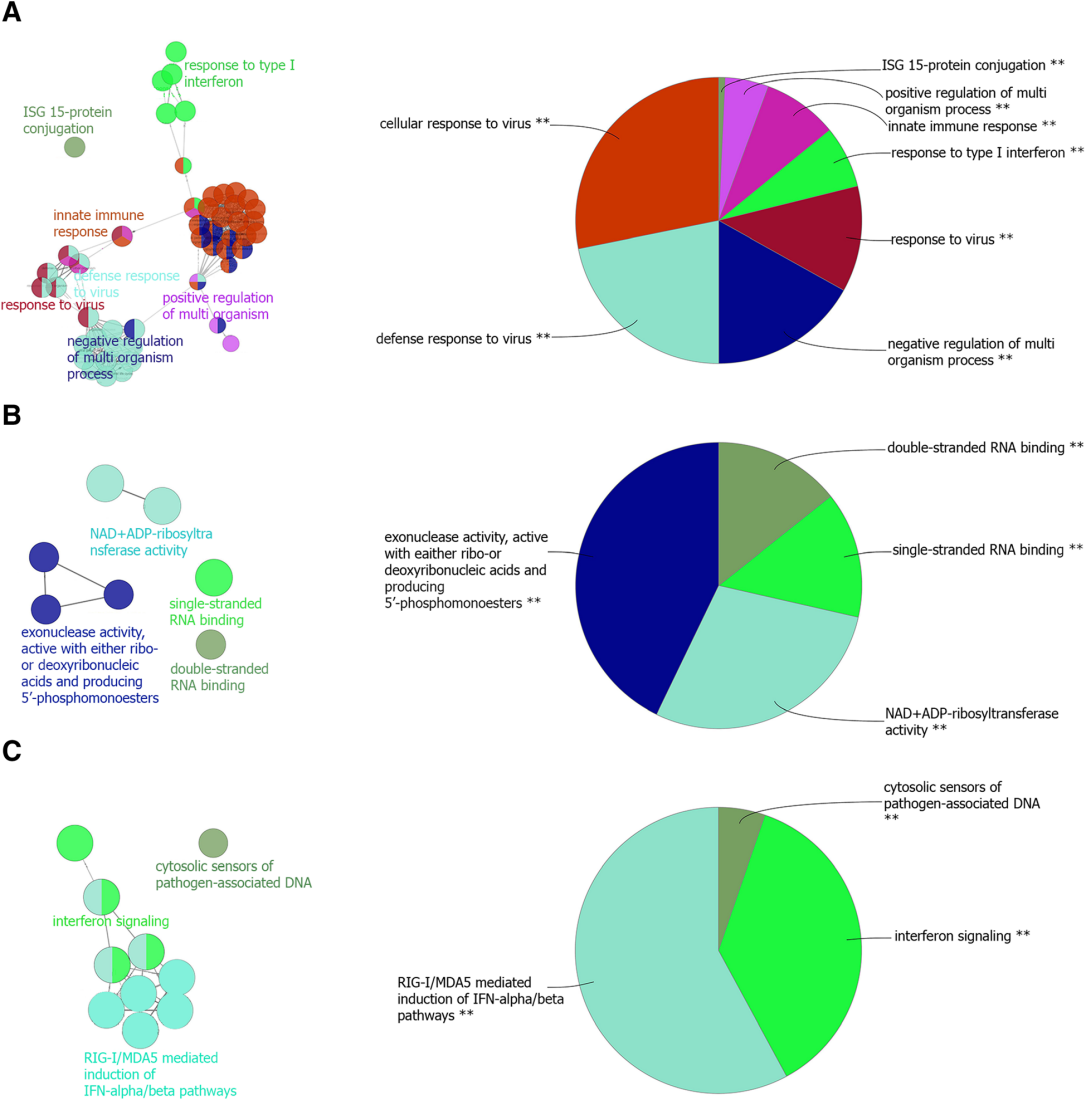
Functional analysis of the white module genes. Over-represented GO/pathway terms were grouped based on kappa statistics. The size of each category within a pie chart represents the number of included terms. Only the most significant GO/terms within groups were labeled. GO/pathway terms are represented as nodes, and the node size represents the term enrichment significance, while the edges represent significant similarity between categories. **a** Representative biological processes interactions among module genes. **b** Representative molecular function interactions among module genes. **c** Representative Ractome analysis interactions among module genes

The white module contained 80 probe sets, which mapped to 63 genes, and had 3160 edges (connections). Interrogation of the cattle protein interaction network database [21] revealed that 38 of 63 genes (60 %) had evidence that they interacted (physically or functionally), i.e. a combined interaction score of more than 600 (Fig. 4 and Additional file 4: Table S4). These results indicated a strong functional concordance between genes included in this module. Through consolidation, significantly over-represented Biological Process (BP) GO terms combined into seven clusters with related functions (Fig. 5a and Additional file 3: Table S3): cellular response to virus (18 genes), defense response to virus (18 genes), negative regulation of multi-organism process (15 genes), response to virus (20 genes), response to type I interferon (18 genes), innate immune response (19 genes), positive regulation of multi-organism process (6 genes) and ISG15-protein conjugation (3 genes). The top over-represented BP GO term in the module was defense response to virus. There was a close similarity between this term and other over-represented BP GO terms in the module (Fig. 5a).

The Molecular Function (MF) GO terms of the genes included in this module were related to regulation of gene expression such as: single-stranded RNA binding, double-stranded RNA binding, NAD+ ADP-ribosyltransferase activity and exonuclease activity, active with either ribo- or deoxyribonucleic acids and producing 5′-phosphomonoesters (Fig. 5b and Additional file 3: Table S3). In addition, this module was highly over-represented for Reactome [20] pathways related to RIG-I/MDA5 mediated induction of IFN-alpha/beta pathways, interferon signaling and cytosolic sensors of pathogen-associated DNA were over-represented (Fig. 5c and Additional file 3: Table S3).

The saddle brown Module had 62 probe sets, which mapped to 53 genes, and contained 1891 edges. Forty two percent of these genes (22 genes) had high interaction scores (interaction score > 600; Additional file 4: Table S4), which highlights the functional connection between genes in this module. This module exhibited several clusters of over-represented BP GO terms related to: defense response to bacterium (20 genes), regulation of inflammatory response (13 genes), leukocyte chemotaxis (10 genes), inflammatory response (15 genes), inflammatory response to antigenic stimulus (5 genes), and leukocyte migration (10 genes). The top over-represented BP GO term in the module was defense response to bacterium (Table 1, Additional file 3: Table S3) and close similarity between this term and the other over-represented GO terms in the module (Additional file 5: Figure S1A) indicate response to bacterium as the main biological function of this module.

Enriched MF GO terms associated with this module were related to cell signaling such as: cytokine activity, non-membrane spanning protein tyrosine kinase activity and RAGE receptor binding (Additional file 3: Table S3 and Additional file 5: Figure S1B). In accordance with these results, this module was highly over-represented for cell surface interactions at the vascular wall as its Reactome pathway (Additional file 3: Table S3 and Additional file 5: Figure S1C).

The light green Module contained 169 probe sets, which mapped to 160 genes that were connected via 14,196 edges. Thirty nine percent of these genes, 62 out of 160 genes, had high protein interaction scores (>600; Additional file 4: Table S4). This module had over-represented BP GO terms related to energy metabolism such as: purine ribonucleoside triphosphate metabolic process (38 genes), generation of precursor metabolites and energy (14 genes) and cristae formation (3 genes) (Additional file 3: Table S3 and Additional file 6: Figure S2A). Enriched MF GO terms associated with this module was related to hydrogen ion transmembrane transporter activity and inorganic cation transmembrane transporter activity (Additional file 3: Table S3 and Additional file 6: Figure S2B). This module was over-represented for the following Rectome pathway terms: citric acid cycle (TCA) and respiratory electron transport, glycolysis, metabolism of nucleotides, processive synthesis on the C-strand of the telomere and *TP53* regulates metabolic genes (Additional file 3: Table S3 and Additional file 6: Figure S2C).

The red module contained 519 probe sets, which mapped to 431 genes, and had 134,421 edges. Just less than half of these genes, 244 out of 519 genes, had high protein interaction scores (>600) (Additional file 4: Table S4) that highlight the functional connection between genes in the module. Gene ontology enrichment analysis revealed cell-cycle as the top over-represented BP GO term in the module. Close similarity between this term and more than 80 % of over-represented BP GO terms in the module (139 out of 160) indicated that cell-cycle was an umbrella process for this module (Additional file 3: Table S3 and Additional file 7: Figure S3A). Molecular function GO terms for this module were related to ATP binding, DNA binding, damaged DNA binding, DNA helicase activity and cyclin-dependent protein serine/threonine kinase regulator activity (Additional file 3: Table S3 and Additional file 7: Figure S3B). This module also displayed a high number of over-represented Reactome pathways related to cell cycle such as chromosome maintenance, mitotic G2-G2/M phases, activation of the pre-replicative complex and mitotic prophase (Additional file 3: Table S3 and Additional file 7: Figure S3C).

The potential function of 133 genes with no previous functional annotation, e.g. no associated/assigned GO terms, was predicted based on functional uniformity among the associated genes (Additional file 8: Table S5). Interestingly, we found four intra-modular hub genes with un-known function: *LOC509513*, *LOC100848208*, *LOC100850151* and *MGC137211* which were located in the light cyan, yellow, blue and brown modules, respectively. Functional analysis of their located module and close interconnectedness (i.e. topological overlap measure (TOM) > 0.01) with known genes (Table 2 and Additional file 8: Table S5) revealed that they are potentially involved in biological functions related to leukocyte activation (*LOC509513*), RNA processing (*LOC100848208*), nucleic acid metabolic process (*LOC100850151*) and organic-acid metabolic process (*MGC137211*). Such highly connected genes should be investigated more closely as they likely to have key regulatory roles in the cattle.

**Table 2:** Functional enrichment analysis of close neighbors (TOM > 0.01) of hub genes with no functional annotation in CGCN^a^

Gene	#	Top BP GO term	*p*-value^b^	Top Reactome pathway	*p*-value^c^
LOC509513	1	T cell activation	1.58E-20	Adaptive Immune System	1.04E-21
	2	T cell aggregation	1.58E-20	Immunoregulatory interactions between a Lymphoid and a non-Lymphoid cell	3.55E-19
	3	lymphocyte aggregation	1.84E-20	Immune System	7.66E-19
LOC100848208	1	RNA processing	8.95E-07	Mitochondrial translation elongation	2.12E-08
	2	mRNA metabolic process	1.87E-04	Mitochondrial translation termination	2.12E-08
	3	mitochondrial translation	2.18E-04	Mitochondrial translation	2.50E-08
LOC100850151	1	RNA processing	3.82E-15	Cell Cycle	8.78E-11
	2	mRNA processing	6.36E-11	Processing of Capped Intron-Containing Pre-mRNA	3.91E-10
	3	mRNA metabolic process	1.56E-10	Cell Cycle, Mitotic	2.79E-08
MGC137211	1	carboxylic acid metabolic process	1.32E-41	Metabolism	4.96E-41
	2	monocarboxylic acid metabolic process	1.05E-23	Biological oxidations	2.20E-18
	3	alpha-amino acid metabolic process	6.84E-20	Metabolism of lipids and lipoproteins	3.72E-18

^a^Just three top over-represented biological process (BP) and Reactome pathways are listed, More information related to these hubs and other un-known genes in the network are provided in Additional file 6: Table S5

^b,c^The *p*-value indicated the probability that a module contains equal or larger number of genes associated with the GO term under a hypergeometric distribution after Bonferroni step-down correction

## Discussion

Gene annotation projects indicate that some protein coding genes in a variety of organisms have no known functionally or have weak functional annotation [22]. Defining the functions of all genes in the genome of an organism is a formidable task. Gene expression data is a valuable resource that can provide possible functional annotation of a gene. Each gene is estimated on average to interact with four to eight other genes and to be involved in 10 biological functions [23]. Gene co-expression analysis provides a framework to study gene function in the context of interactions derived from multiple data sources and integrated into a global interactome. With emphasis on cattle the application of RNA-sequencing has paved the way for transcriptome analysis of cattle in recent years in various experimental conditions [24–26]. For the purpose of genome-wide co-expression analyses, a comprehensive catalogue of experimental conditions from RNA-seq studies is still incomplete. Nevertheless, historical microarray data have provided a basis for genome-wide co-expression studies in cattle. In this study, cattle condition independent gene co-expression networks were generated using the large number of publicly available high-quality microarray chips from either NCBI GEO [27] or EBI ArrayExpress [28]. The hypothesis of this study was that genes with similar pattern of accumulation across a vast array of tissues and experimental conditions are likely to encode proteins with related functions [14]. The first attempt to construct a genome wide gene co-expression network has been made by Lee et al. [29]. They presented a large-scale analysis of mRNA co-expression based on 60 large human data sets and explained how the large body of accumulated microarray data can be exploited to increase the reliability of inferences about gene function. Since then, several attempts have been made to construct massive scale gene co-expression network as a source of functional annotation in many species from yeast to human [12–16].

WGCNA [18], a powerful ‘guilt-by-association’-based method, was used to construct CGCN. Several measures can be used to define correlation coefficient in correlation networks such as Pearson correlation, Spearman correlation and Biweight midcorrelation [9]. The Pearson correlation is sensitive to outlying observations and it just considers the linear relations between variables. While Spearman correlations protect against outliers and can account for non-linear relations. It is however overly conservative in many applications [9]. In this study, we used Biweight midcorrelation for network construction which combines the advantages of both the Pearson (relatively high power) and Spearman correlations (relatively high robustness) [30].

WGCNA is based on the concept of a scale-free network. Metabolic networks in all organisms have been suggested to be scale-free networks [18], and scale-free network phenomena have been observed in many empirical studies [31–33]. Scale-free networks are extremely heterogeneous, and their topology being dominated by a few highly connected nodes that link the rest of the less connected nodes to the system [9]. The main property of scale-free networks is their remarkable tolerance against attacks of randomly selected nodes but not against directed removals of central nodes (hubs) [18]. These hub nodes can be detected using nodes connectivity in the whole network (whole network hubs) or in the subnetworks of the main network (intra-modularhubs) [9]. Intra-modularconnectivity has been found to be an important complementary node screening variable for finding biologically important genes [18]. They argued that while whole-network connectivity is important in many context, nodes important for particular functions in large, complex networks are often not among the whole-network hubs [18]. Based on these results, intra-modularconnectivity was used to detected hub genes in CGCN.

Constructing a gene co-expression network and naturally partitioning the network into modules, provided a systems-level understanding of the gene modules that coordinate multiple biological processes to carry out specific biological functions [13]. The effectiveness of our approach is best illustrated by correspondence of these computational modules with actual biological entities. Most of gene interactions found in each module were also supported with protein interaction data (physical or functional interactions) from String database [21].

The white module had several close interconnected over-represented GO terms and Reactome pathways related to immune response to virus (Fig. 5). This module was also enriched for ISG15-protein conjugation BP GO terms, which were associated with *ISG15*, *UBA7* and UBE2L6. ISG15 ubiquitin-like modifier gene (*ISG15*) encodes for an interferon induced ubiquitin like (UbL) protein, which plays a key role in the innate immune response to viral infection either via its conjugation to both host and viral proteins (ISGylation) or via its action as a free or unconjugated protein [34]. The ISGylation process requires three sequential reaction steps: activation, conjugation and ligation, which are performed by E1-E3 enzymes, respectively [35]. The other two genes, *UBA7* and *UBE2L6*, encode for ubiquitin/*ISG15* activating and conjugating enzymes, respectively [36]. Another gene in the module, *HERC6*, has ligase activity and is involved in the UbL conjugation pathway [36]. *HERC6* has been reported to be important in the antiviral response [37], where it functions as the main E3 ligase for global *ISG15* conjugation in mouse cells [38]. The expression change and direct regulation of HERC6 and Interferon-Simulated Genes (ISGs) by interferon *Tau* (IFNT) has been shown in cattle endometrium [39]. Interferon *Tau* shows antiproliferative effects and antiviral activities that have less toxicity than the other type-I IFNs [40]. Ubiquitin specific peptidase (*USP18*) had the highest intra-modularconnectivity and is the hub node for the white module (Additional file 2: Table S2). This gene has *ISG15*-specific protease activity, i.e. it removes *ISG15* from ISGylated proteins [41] as evidenced by its associated MF GO term [19]. *USP18* protein highly interacts (i.e. combined interaction score > 600) with 40 % of the proteins encoded in the white module (25 out of 63 proteins) (Fig. 4 and Additional file 4: Table S4). For example, a combined interaction score > 600 meant that connection between two proteins ranked in the top 90 percentile combined scores in the String database [21] (Additional file 9: Figure S4A). These results might indicate its close functional relations with the other genes included in the white module. The function of this gene is crucial for proper cellular balance of *ISG15*-conjugated proteins [41]. In addition, *USP18* has a major role in the regulation of signal transduction pathways triggered by type I interferons (IFNs) [36], which play a central role in the antiviral innate immune response of vertebrates [35].

Regulation of gene expression is determined in large part by the activity of transcriptional activator proteins. Also, transcriptional regulation enables cells to respond to environmental cues such as viral infection [42]. Two members of interferon regulatory factors (IRFs) gene family, IRF7 and IRF9, were included in the white module. IRFs transcription factors (TFs) regulate IFNs gene transcription and protein production [43] and have a well-known activity against pathogenic infections in several species [44]. In cattle, the antiviral activity of *IRF7* and *IRF9* has been reported to be associated with both Bovine Herpesvirus1 [45] and Foot-and-Mouth Disease Virus [46, 47] infection. High connectivity (TOM > 0.01) between the module hub node and these TFs indicate that they are potentially co-regulated. For example, a TOM score > 0.01 meant that connection between two genes ranked in the top 99 percentile connectivity across networks (Additional file 9: Figure S4B).

The saddle brown module was highly enriched for several BP GO terms related to response to bacterium (Additional file 5: Figure S1A). In addition, this module had over-represented MF GO terms and Reactome pathways related to different aspects of cellular surface interactions involved in immune response (Additional file 5: Figure S1A, B). Individual cells monitor their surrounding environment and react to extracellular challenges that require adaptation or threaten viability [48]. The plasma membrane forms a barrier between a cell and its surroundings and participates in the initial response to biological attack [48]. Cytokines, important mediators of immune responses, are secreted by immune cells in response to pathogenes, and bind to specific membrane receptors, which then signal the cell via second messengers, often tyrosine kinases, to alter cellular activity, e.g. gene expression [49]. Four genes with cytokine activity were included in the module: *IL10*, *IL1B*, *IL1RN* and *PF4*. The antibacterial activity of these well-known genes with the highest quality annotation scoring in the UniProt database [50], have been reported in response to several bacterial infections in cattle [51–53]. The saddle brown module hub gene, *S100A9* plays a prominent role in the regulation of inflammatory processes and immune response [36]. This gene can induce neutrophil chemotaxis, adhesion and increase the bactericidal activity of neutrophils by promoting phagocytosis [54]. It has antibacterial activity via chelation of Zn^2+^, which is essential for bacterial growth [54]. In addition, *S100A9* acts as an alarmin or a danger associated molecular pattern (DAMP) and can stimulate innate immune cells via binding to pattern recognition receptors such as Toll-Like Receptor 4 (TLR4) [36].

The light yellow module had several over-represented GO terms and Reactome pathways related to different aspects of energy metabolism (Additional file 6: Figure S2). In addition, this module was highly enriched for cristae formation as BP GO term (Additional file 6: Figure S2A). The unbiased studies on knockout mice revealed that telomere dysfunction is associated with impaired mitochondrial biogenesis and energy production [55]. Despite the over-representation of the GO term, *TP53* Regulates Metabolic Genes pathway, in the light green module, the *TP53* gene was not included in the module. This gene was included in the yellow module that showed high enrichment for GO terms related to gene expression and RNA processing (Additional file 3: Table S3). Closer inspection of nine genes in TP53 regulates metabolic genes pathway (*COX16*, *COX5A*, *HDAC1*, *LAMTOR1*, *MED27*, *TFDP2*, *TXNRD1*, *YWHAB* and *YWHAQ*) revealed tight connectivity (TOM > 0.01) between the probe sets that mapped to these genes and *TP53*. This result indicates that TP53 might be an intermediate node between the yellow and light green modules. COX5A gene had the highest connectivity in the module and considered as an intra-modular hub node. This gene encodes for mitochondrial Cytochrome c oxidase subunit 5A, which is the heme A-containing chain of cytochrome c oxidase, the terminal oxidase in mitochondrial electron transport and has a key role in cell energy production [36].

The red module had over-represented GO terms and pathways related to cell cycle process (Additional file 7: Figure S3). The typical eukaryotic cell cycle is divided into four phases: two gap phases (G1 and G2); a synthesis phase (S), in which the genetic material is duplicated; and an M phase, in which mitosis partitions the genetic material and the cell divides [56]. The regulation of gene expression is an important component of cell cycle control [57]. Cyclins are one of the main cell cycle regulatory proteins that control the progression of cells through the cell cycle by activating cyclin-dependent kinase (CdK) enzymes [58]. Ten cyclin genes included in the module were: *CCNA2*, *CCNB1*, *CCNB2*, *CCNE1*, *CCNE2*, *CCNF*, *CDKN1A*, *CDKN2C*, *CKS1B* and *CKS2*. These genes regulate different cell cycle phases such as G1/S (*CCNE1*, *CCNE2* and *CDKN2C*), G2/S (*CDKN1A*), G2/M (*CCNA2* and *CDKN1A*) and cell division (*CCNF*, *CKS1B*, *CKS2*, *CCNB1* and *CCNB2*) [36]. *CDCA8* gene had the highest connectivity in the red module and considered as the intra-modular hub node (Additional file 2: Table S2). This gene is a component of the chromosomal passenger complex (CPC), a complex that acts as a key regulator of mitosis [36]. The CPC complex has essential functions at the centromere in ensuring correct chromosome alignment and segregation and is required for chromatin-induced microtubule stabilization and spindle assembly [36].

The fact that functionally related genes are connected together in co-expression networks provides evidence for prediction of the cellular roles for hypothetical genes based on a GBA principle [11]. Neighborhood (genes that are highly connected to a given set of genes) analysis based on TOM can be used as a powerful tool for this purpose. Briefly, two genes have a high topological overlap if they connect and disconnect the same genes. The potential cellular roles of 132 functionally unknown cattle genes including four unknown hub genes were predicted using neighborhood analysis (Additional file 6: Table S5) based on GBA principle. There were weak sequence similarities between these potential genes and known genes in orthologous species. The results of this study might be used as a new insight for possible biological function of these potential genes.

Genes with little to no associated functional information generally have no gene symbol and so are automatically assigned an identifier such as LOC533597. Gene nomenclature, i.e. the scientific naming of genes, tries to standardized representation of genes within an organism, but not necessarily between organisms, based on the biological process or pathway in which they are involved. Although the results of the current study cannot be used directly for nomenclature purposes as they have no supporting biological information, they provide a rich resource for experimental biologists to begin to define the real biological function and thereby helping to assign gene symbols to such genes.

## Conclusions

In summary, these analyses indicate that the cattle gene co-expression network and corresponding regulons provides rich information for experimental biologists to design experiments, interpret experimental results, and develop novel hypotheses on gene function in cattle. Combinatorial approaches that integrate multiple omics findings will provide an important resource that should lead to the elucidation of molecular mechanisms underlying traits of interest in cattle.

## Methods

### Microarray data analysis

CEL files for 1470 publicly available *Affymetrix* Genechip Bovine Genome Array (Bos taurus) were downloaded from either NCBI GEO [27] or EBI ArrayExpress [28] (Additional file 1: Table S1). Arrays from individual experiments were preprocessed as briefly described; expression levels were summarized, log2 transformed and normalized using the robust multichip analysis algorithm (RMA) as implemented in the R Affy package [59]. Quality tests were performed on the normalized array data using the Bioconductor arrayQualityMetrics package [60]. Arrays that failed all three outlier tests (i.e. Distances between arrays, Boxplots and MA plots) were excluded from further analyses. The annotation information of the Affymetrix Genechip Bovine Genome Array was obtained from the GPL2112 microarray platform (August 2014) [27]. Microarray probe sets were mapped to Bos taurus UMD 3.1.1 genome assembly using AffyProbeMiner [61] with December 2014 release of Bos taurus genome annotation as reference [62]. The control probes from the *Affymetrix* Genechip Bovine Genome Array were removed from the sample data. Probe-set IDs that did not map to an Entrez gene ID or Probe-set IDs that mapped to multiple Entrez gene IDs were discarded. The parametric Bayes Combat algorithm [63] was used to re-scale the expression intensity and remove experimental batch effects.

### Weighted Gene Co-expression Network Analysis (WGCNA)

The WGCNA R package [18] was used to identify network modules from normalized gene expression values. Briefly, an adjacency matrix was formed with elements *r*_*ij*_, which were the Biweight midcorrelation coefficient [9] between expression values of probe sets i and j. A connectivity measure (k) per probe set was calculated by summing the connection strengths with other probe sets. Subsequently as described by Zhao et al. [23], the adjacency matrix was replaced with the weighted adjacency matrix based on the β parameter with a scale‑free topology criterion. The goodness of fit of the scale-free topology was evaluated by the Scale-Free Topology Fitting Index (*R* ^2^), which was the square of the correlation between log (*p* (*k*)) and log (*k*). A *β* coefficient of seven with *R* ^2^ of 0.9 was used to develop a weighted adjacency matrix. The weighted adjacency matrix was used to then develop the topological overlap matrix (TOM) as described by Langfelder et al. [18]. The TOM reflects the relative interconnectivity between two genes based on their degree of shared neighbors across the whole network [18]. Dynamic Tree Cut algorithm [64], which utilized a gene tree dendrogram that was developed based on TOM-based dissimilarity (1-TOM) using hclust algorithm [65], was used to place probe sets into modules. Within the cutreeDynamic function, deep split was set to two and minimum module size was set to 25. Similar modules were merged based on their eigengenes similarities using mergeCloseModules function and height cut of 0.2. All other WGCNA parameters remained at their default settings.

### Protein interaction information

Potential interaction between genes included in each module were evaluated with the protein interaction network (v10) from String database [21]. String uses eight major sources of interaction/association data (neighborhood, fusion, co-occurrence, co-expression, experimental, database and text mining) to define interaction between proteins using a probabilistic confidence score [21]. The combined score [21] of all these available resources were used to estimate the interaction strength between proteins. If the interaction between two genes was based on more than one protein-protein interaction, the interaction scores were averaged using a custom R script.

### Gene ontology and pathway enrichment analysis

To decipher the potential mechanism of action of detected modules, ClueGO [66], a widely used Cytoscape [67] plugin, was applied to identify biological interpretation of functional modules in the network. The latest update of gene ontology annotation database (GOA) [19] and Reactome pathway database [20] (released November 2015) were used in the analysis. Genes included on Affymetrix Bovine Genechip Array were used as background. Ontologies were designated as biological processes, molecular function and Reactome pathways. The GO tree interval ranged from 3 to 20 with the minimum number of genes per cluster set to three. Term enrichment was tested with a right-sided hyper-geometric test that was corrected for multiple testing by the Bonferroni step-down method [68]. Only GO/pathway terms that were significantly enriched (*p*-value ≤ 0.05) were included in the analysis. Kappa statistics were used to link and grouping of the enriched terms and functional grouping of them as described in [66]. The minimum connectivity of the pathway network (kappa score) was set to 0.4 units.

## Additional files

Additional file 1: Table S1. Microarray datasets used in this study. (XLSX 152 kb)
Additional file 2: Table S2. Module membership and connectivity of 16,608 probe sets used in this study. (XLSX 980 kb)
Additional file 3: Table S3. Over-represented GO/Pathway terms in the co-expressed modules. (XLSX 287 kb)
Additional file 4: Table S4. String [21] protein interaction information of four selected modules in CGCN. (XLSX 1177 kb)
Additional file 5: Figure S1. Functional analysis of the red module. Over-represented GO/pathway terms were grouped based on kappa statistics. The size of each category within a pie chart represents the number of included terms. Only the most significant GO/terms within groups were labeled. GO/pathway terms are represented as nodes, and the node size represents the term enrichment significance, while the edges represent significant similarity between categories. (A) Representative biological processes interactions among module genes. (B) Representative molecular function interactions among module genes. (C) Representative Ractome analysis interactions among module genes. (PDF 2606 kb)
Additional file 6: Figure S2. Functional analysis of the light green module genes. Over-represented GO/pathway terms were grouped based on kappa statistics. The size of each category within a pie chart represents the number of included terms. Only the most significant GO/terms within groups were labeled. GO/pathway terms are represented as nodes, and the node size represents the term enrichment significance, while the edges represent significant similarity between categories. (A) Representative biological processes interactions among module genes. (B) Representative molecular function interactions among module genes. (C) Representative Ractome analysis interactions among module genes. (PDF 2605 kb)
Additional file 7: Figure S3. Functional analysis of the red module genes. Over-represented GO/pathway terms were grouped based on kappa statistics. The size of each category within a pie chart represents the number of included terms. Only the most significant GO/terms within groups were labeled. GO/pathway terms are represented as nodes, and the node size represents the term enrichment significance, while the edges represent significant similarity between categories. (A) Representative biological processes interactions among module genes. (B) Representative molecular function interactions among module genes. (C) Representative Ractome analysis interactions among module genes. (PDF 3753 kb)
Additional file 8: Table S5. Functional enrichment analysis of close neighbors (TOM > 0.01) of 133 un-annotated genes in CGCN. (XLSX 479 kb)
Additional file 9: Figure S4. (A) Frequency of combined interaction scores from the String database [21] and (B) Frequency of TOM connectivity in BGCN. (PDF 648 kb)

## Abbreviations

BP: Biological process
CdK: Cyclin-dependent kinase
CGCN: Cattle gene co-expression network
CPC: Chromosomal passenger complex
DAMP: Danger associated molecular pattern
EBI: European Bioinformatics Institute
G: Gap phase
GBA: Guilt-by-association
GCA: Gene co-expression analysis
GEO: Gene Expression Omnibus
GO: Gene ontology
GOA: Gene ontology annotation database
IFNT: Interferon *Tau*
IRFs: Interferon regulatory factors
ISGs: Interferon-Simulated Genes
k: Connectivity measure
MF: Molecular function
NCBI: National Center for Biotechnology Information
*R*^2^: Scale Topology Fitting Index
S: Synthesis phase
TCA: Citric acid cycle
TFs: Transcription factors
TLR4: Toll-Like Receptor
TOM: Topological overlap matrix
UbL: Ubiquitin like
WGCNA: Weighted Gene Co-expression Network Analysis
