# Supplementary figures and images for: Large-scale gene co-expression network as a source of functional annotation for cattle genes
Source: BMC Genomics. 2016 Nov 2;17:846. doi: 10.1186/s12864-016-3176-2 (PMC5094014; doi:10.1186/s12864-016-3176-2)

A

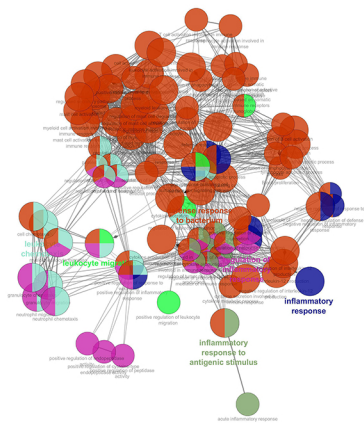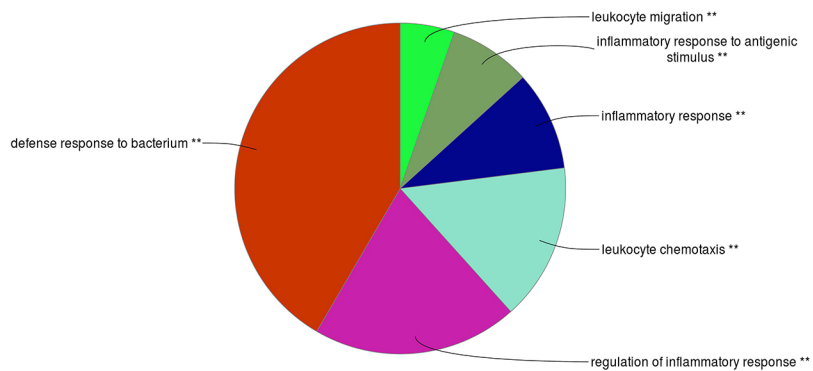

B

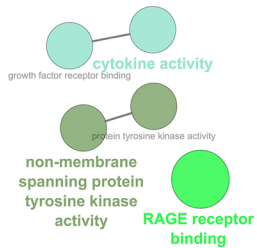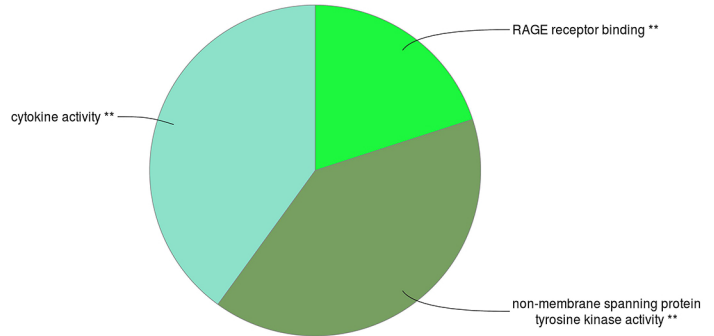

C

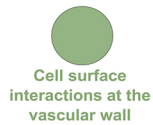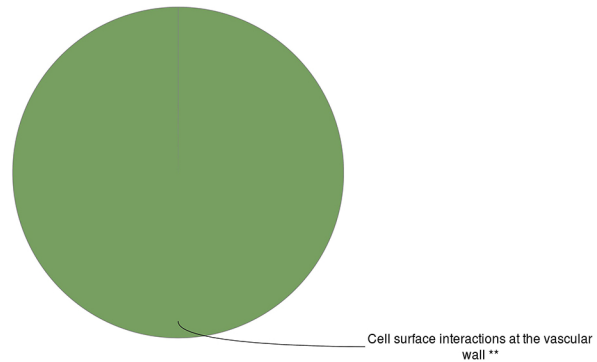

Supplement: Additional file 5: Figure S1. — Functional analysis of the red module. Over-represented GO/pathway terms were grouped based on kappa statistics. The size of each category within a pie chart represents the number of included terms. Only the most significant GO/terms within groups were labeled. GO/pathway terms are represented as nodes, and the node size represents the term enrichment significance, while the edges represent significant similarity between categories. (A) Representative biological processes interactions among module genes. (B) Representative molecular function interactions among module genes. (C) Representative Ractome analysis interactions among module genes. (PDF 2606 kb) [file 12864_2016_3176_MOESM5_ESM.pdf]

A

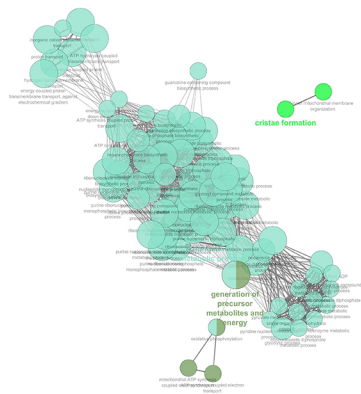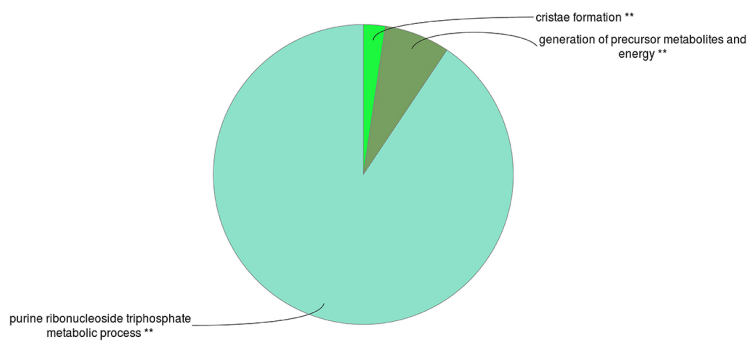

B

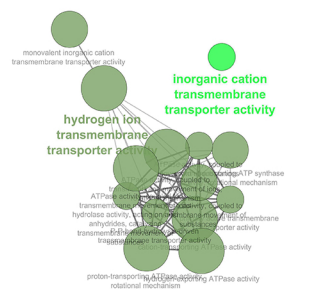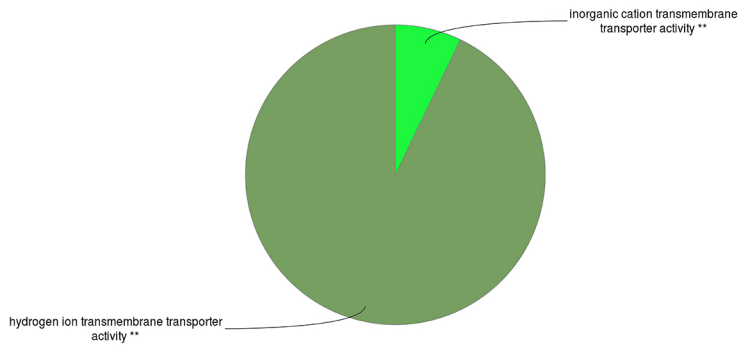

C

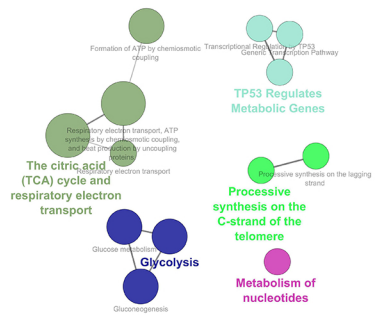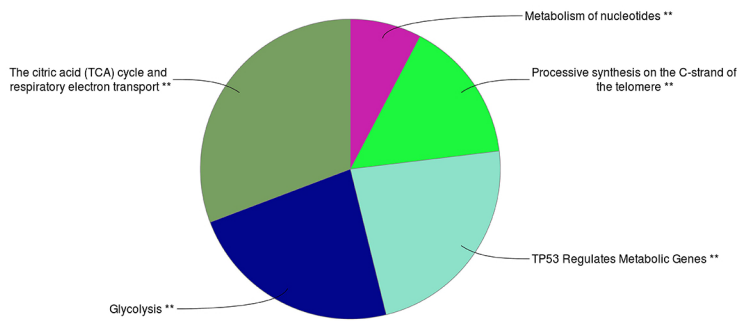

Supplement: Additional file 6: Figure S2. — Functional analysis of the light green module genes. Over-represented GO/pathway terms were grouped based on kappa statistics. The size of each category within a pie chart represents the number of included terms. Only the most significant GO/terms within groups were labeled. GO/pathway terms are represented as nodes, and the node size represents the term enrichment significance, while the edges represent significant similarity between categories. (A) Representative biological processes interactions among module genes. (B) Representative molecular function interactions among module genes. (C) Representative Ractome analysis interactions among module genes. (PDF 2605 kb) [file 12864_2016_3176_MOESM6_ESM.pdf]

A

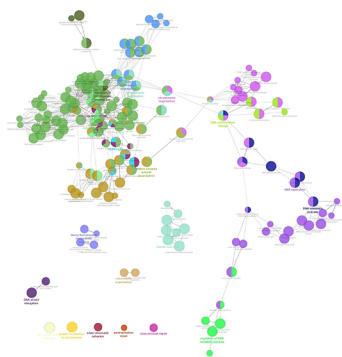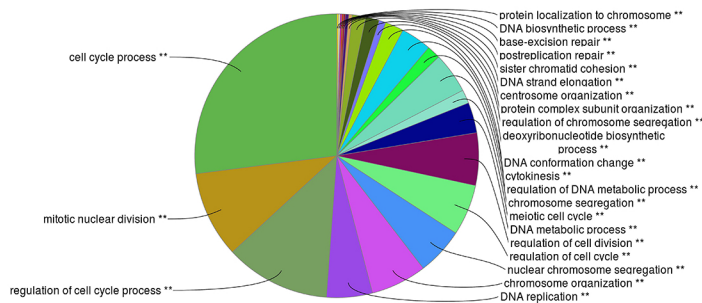

B

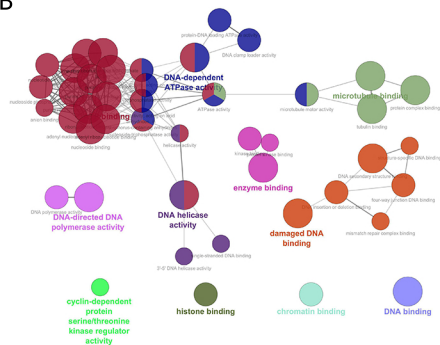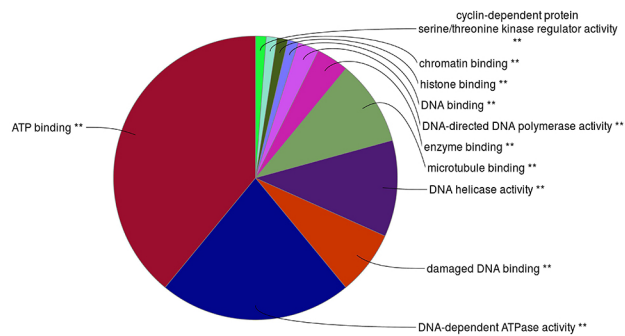

C

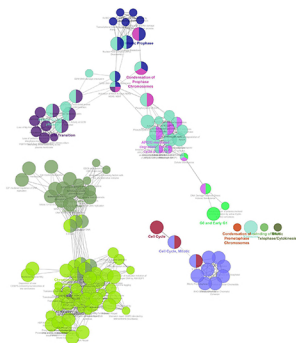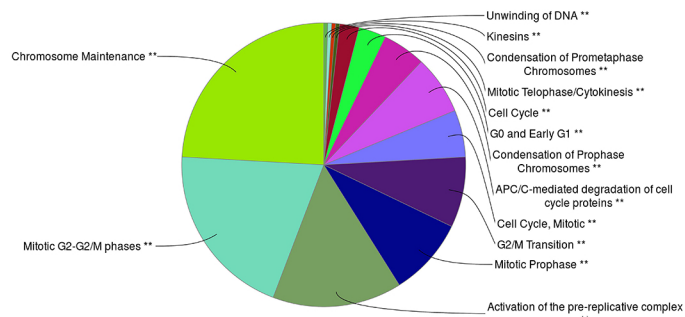

Supplement: Additional file 7: Figure S3. — Functional analysis of the red module genes. Over-represented GO/pathway terms were grouped based on kappa statistics. The size of each category within a pie chart represents the number of included terms. Only the most significant GO/terms within groups were labeled. GO/pathway terms are represented as nodes, and the node size represents the term enrichment significance, while the edges represent significant similarity between categories. (A) Representative biological processes interactions among module genes. (B) Representative molecular function interactions among module genes. (C) Representative Ractome analysis interactions among module genes. (PDF 3753 kb) [file 12864_2016_3176_MOESM7_ESM.pdf]

A

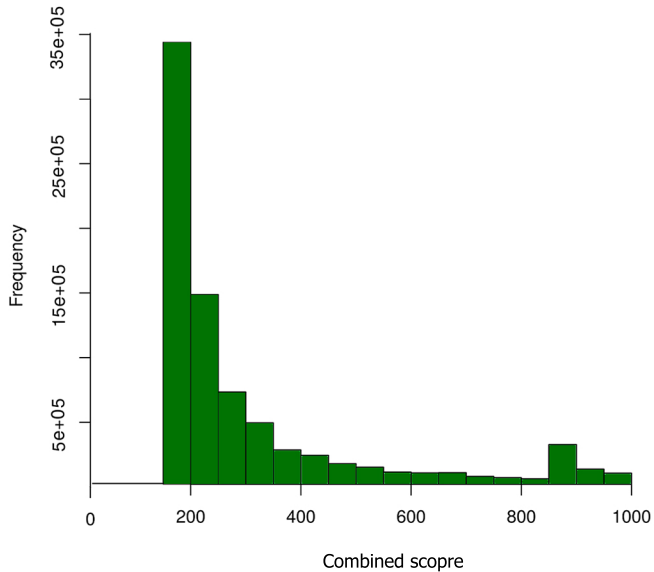

B

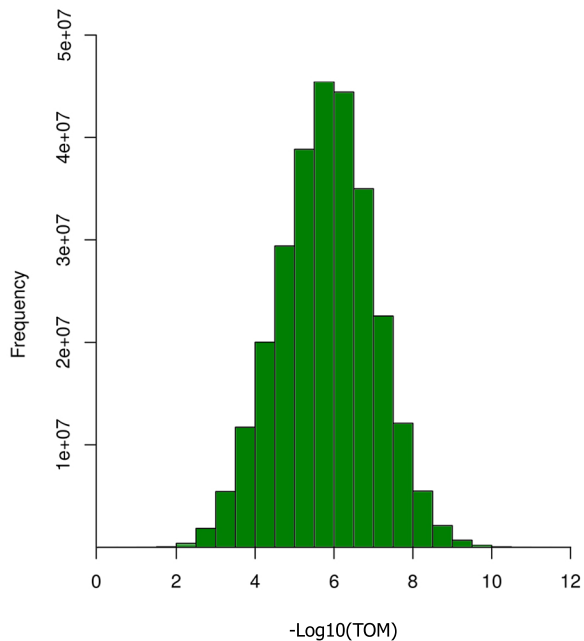

Supplement: Additional file 9: Figure S4. — (A) Frequency of combined interaction scores from the String database [21] and (B) Frequency of TOM connectivity in BGCN. (PDF 648 kb) [file 12864_2016_3176_MOESM9_ESM.pdf]
